# Recent advances in small molecule LpxC inhibitors against gram-negative bacteria (2014–2024)

**DOI:** 10.3389/fmicb.2025.1541379

**Published:** 2025-02-10

**Authors:** Pengpeng Ji, Meng Ma, Xiaoyue Geng, Jian Zhang

**Affiliations:** School of Pharmacy, Shandong Second Medical University, Weifang, Shandong, China

**Keywords:** LpxC inhibitors, gram-negative bacteria, multidrug-resistant (MDR), CHIR-090, TP 0586532

## Abstract

In 2024, WHO added multiple multidrug-resistant (MDR) Gram-negative bacteria to the bacteria priority pathogens list, and the continued increase in MDR Gram-negative bacteria poses a serious threat to public health. Uridine diphosphate-3-*O*-(hydroxymyristoyl)-*N*-acetylglucosamine deacetylase (LpxC) is a metalloenzyme cofactored with zinc ions, which is a key enzyme in the synthesis of outer membrane lipid A in Gram negative bacteria. LpxC is highly conserved and homologous among different Gram-negative bacteria, which makes LpxC a promising target against multidrug-resistant Gram-negative bacteria. Since the first report of the arazoline LpxC inhibitor L-573, 655, a large number of small molecule LpxC inhibitors against Gram-negative bacteria have been synthesized and tested, such as TU-514, CHIR-090, ACHN-975 and TP0586532. However, only ACHN-975 entered clinical phase I trials and was discontinued due to safety concerns, so far none of the LpxC inhibitors are available. This paper mainly focuses on the structure optimization, conformational relationship and animal toxicity of small molecule LpxC inhibitors over the past 10 years, especially in the last 5 years, in order to provide ideas for the development and clinical research of LpxC inhibitors.

## 1 Introduction

Multidrug-resistant organisms (MDROs) are the main causative agents of nosocomial infections in hospitals and have become a major challenge in global public health (Tinelli et al., 2021). Four of the six common multidrug-resistant bacteria are Gram-negative in clinical practice (Zoghlami et al., 2023). The last antibiotic with a novel mechanism of action against Gram-negative bacteria was approved by FDA nearly 60 years ago (Prasad et al., 2022). Therefore, the research and development of antibiotics with novel structures and unexploited targets is an urgent need for the treatment of MDR bacterial pathogens.

**Figure 1:**
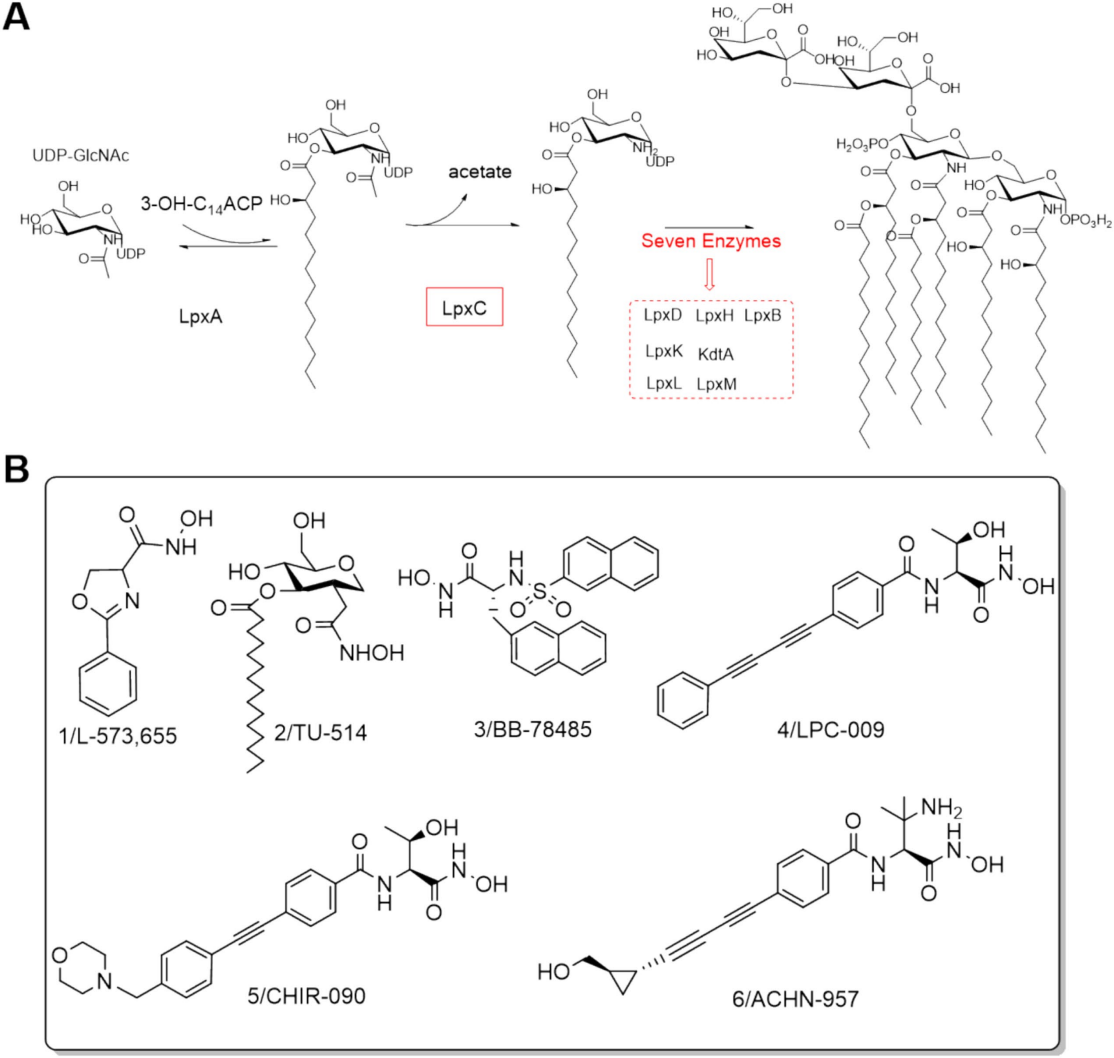
**(A)** Lipid A biosynthesis; **(B)** Structures of LpxC inhibitors before 2014.

Lipopolysaccharide (LPS), as a major component of the outer membrane of the characteristic structure of Gram-negative bacteria, triggers a very strong and even lethal immune response in the host and is indispensable for the survival of Gram-negative bacteria (Matsuura, 2013). Lipid A, the main toxic component of LPS, is responsible for the proper assembly and anchoring of LPS, and inhibition of its synthesis can lead to Gram-negative bacteria death (Ulaganathan et al., 2007). As shown in Figure 1A, UDP-3-O-acyl-N-acetylglucosamine deacetylase (LpxC) is an essential enzyme for the second (irreversible) step of the reaction in the lipid-like A biosynthetic pathway (Langklotz et al., 2012; Zhang et al., 2012). Comparison of gene sequences revealed that LpxC has high homology among Gram-negative bacteria and has no genetic homology with mammals, including humans (Barb and Zhou, 2008). Thus, the development of LpxC inhibitors is a very promising strategy for the research of anti-Gram-negative bacteria drugs.

The discovery of aryloxazoline derivative L-573,655 (Figure 1B) offered a new avenue for the design and development of LpxC inhibitors (Onishi et al., 1996). In 1999, Jackman et al. developed the substrate-based hydroxamate LpxC inhibitor TU-514 (Figure 1B) by integrating the hydroxamic acid into the tetrahydropyran ring (Jackman et al., 2000; Jackman et al., 1999). Subsequently, numerous potent Lpxc inhibitors were reported, such as BB-78485, LPC-009, CHIR-090 and ACHN-975 (Figure 1B) (Barb et al., 2009; Brown et al., 2012; Caughlan et al., 2012; Clements et al., 2002; Cole et al., 2011; Lee et al., 2013; McClerren et al., 2005). Notably, ACHN-975 displayed better antibacterial activities against both susceptible and resistant *P. aeruginosa* than those of the clinically used first- and second-line antimicrobial drugs, such as tobramycin, ciprofloxacin, ceftazidime, imipenem, and mucomycin. ACHN-975 was the first drug to enter clinical phase I trials, but the trials were stopped due to its off-target side effects (González-Bello, 2017; Krause et al., 2019).

This review mainly focuses on the structural optimization, structure–activity relationship, animal toxicity, and future research directions of LpxC small molecule inhibitors from 2014 to 2024, in order to provide ideas for the development and clinical research of LpxC inhibitors.

## 2 Structural insights of LpxC

**Figure 2:**
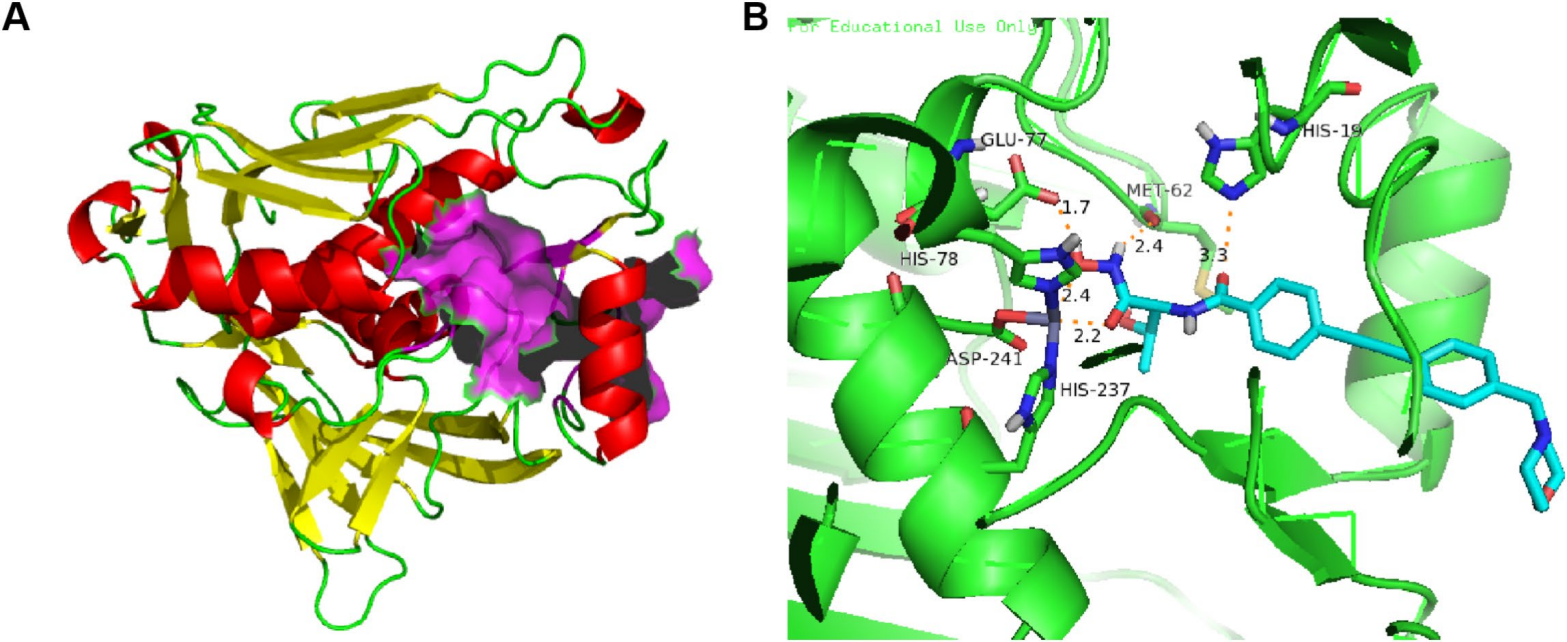
**(A)** Protein structure of *P. aeruginosa* LpxC (PDB code: 7DEM; The *α*-helixes are shown in red; The *β*-structures are shown in yellow; The LpxC inhibitor binding region is shown in purple); **(B)** The binding mode of CHIR-090 in complex with *P. aeruginosa* LpxC (PDB code: 7DEM; Key amino acid residues are shown in yellow).

Extensive X-ray crystallographic studies of LpxC homologs from several bacteria have been performed, as shown in Figure 2A. LpxC contains two similarly folded structural domains, each containing two layers of secondary structure units. A layer of *α*-helices is stacked on the primary *β*-fold, which consists of five chains of 4–6 residues mixed in parallel and antiparallel orientations (Coggins et al., 2003; Kumar Pal and Kumar, 2023). The β-folding of structural domain I is severely distorted, whereas the β-folding of structural domain II is essentially flat. The insertion region of structural domain I has a small three-stranded antiparallel β-fold (βa, βb, and βc), whereas the insertion of structural domain II has a β-α-α_L_-β motif, with the two insertion fragments roughly perpendicular to the main β-fold (Coggins et al., 2003). The two insertion regions are located on the same side of the molecule and together form the active site, establishing a conserved hydrophobic channel that can bind fatty acids (Clayton et al., 2013).

The active-site in *P. aeruginosa* LpxC has an atypical binding motif consisting of two histidines (His 78 and His 237) and an aspartic (Asp 241) acid, which may be advantageous in selectivity of inhibitor for other Zn^2+^-dependent enzymes (Hernick et al., 2010; Jackman et al., 1999). As shown in Figure 2B, the amide carbonyl O atom of CHIR-090 threonine is 3.3 Å away from the imidazole group of (His19) and forms a hydrogen bond with it. The carboxylic acid carbonyl oxygen atom of Glu77 is 1.7 Å away from the hydroxyl group of hydroxamic acid and forms a hydrogen bond. Met62 well localizes the amine group of hydroxamic acid through hydrogen bonding forces. The biphenylacetylene portion of CHIR-090 is inserted into the LpxC hydrophobic channel near the active site, and within the hydrophobic channel, the aromatic rings of CHIR-090 are not coplanar. The morpholine ring adopts a boat conformation and lies flat, with one side against the LpxC surface and the other side exposed to solvent. The morpholine ring does not appear to be coordinated by hydrogen bonding, although potential hydrogen bonds accept O and N atoms.

## 3 Pharmacophore of LpxC inhibitors (CHIR-090 as an example)

Almost all representative LpxC inhibitors contain a zinc ion chelat group (ZBG), a linker and a hydrophobic fragment. In addition, some compounds also have UDP binding site and tail modifier group. To fully illustrate the pharmacophore of LpxC inhibitors, we take CHIR-090 as an example to discuss in detail.

**Figure 3:**
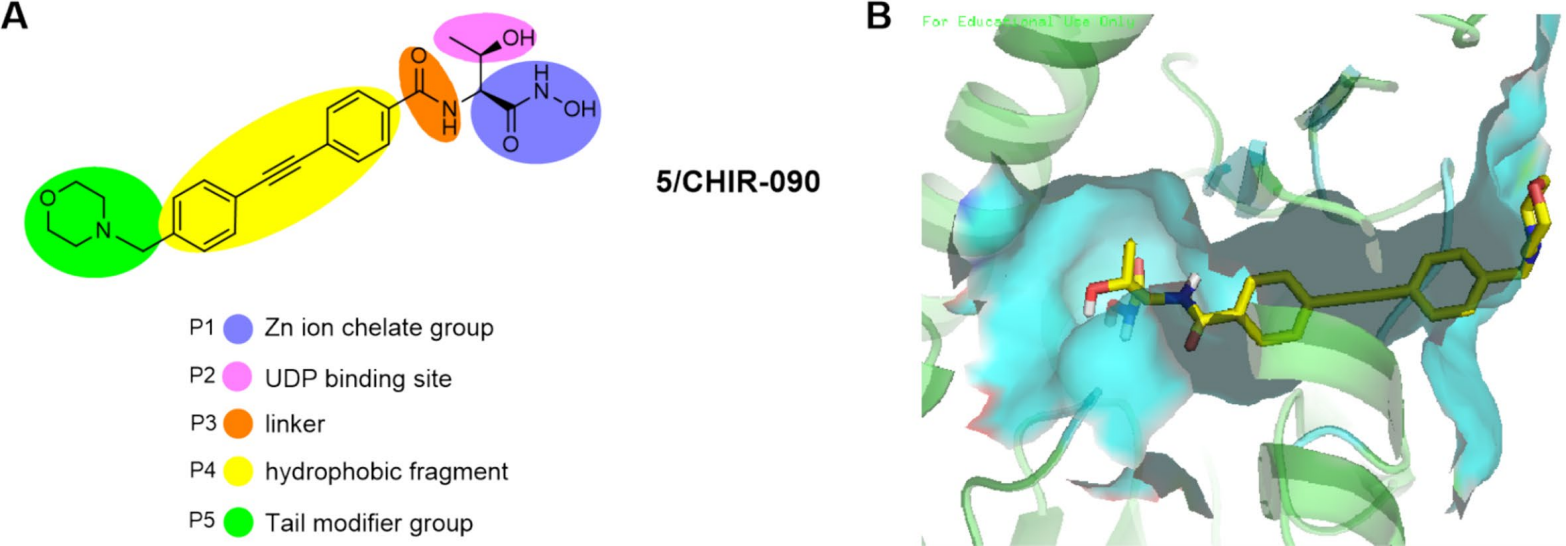
**(A)** Pharmacophore of LpxC inhibitors (e. g. CHIR-090); **(B)** The binding mode of CHIR-090 in complex with *P. aeruginosa* LpxC. (PDB code: 7DEM).

As shown in Figures 3A,B, the structure of CHIR-090 could be divided into five parts, P1: zinc ion chelating group; P2: UDP pocket binding site with different substituents having different effects on antimicrobial activity; P3: linker connecting hydrophobic and chelating groups; P4: for the hydrophobic fragment occupying the LpxC enzyme; P5: terminal group (solvent-exposed region), which generally has little effect on the antimicrobial activity and mainly improves the molecular physicochemical or metabolic properties of the molecule. Based on the above pharmacophore model, together with the three-dimensional structure of the LpxC enzyme, a lot of more potent LpxC inhibitors have been identified.

## 4 LpxC inhibitors from 2014 to 2024

### 4.1 Hydroxamic acid LpxC inhibitors

#### 4.1.1 Difluoromethyl-L-threonine hydroxamic acid LpxC inhibitors

**Figure 4:**
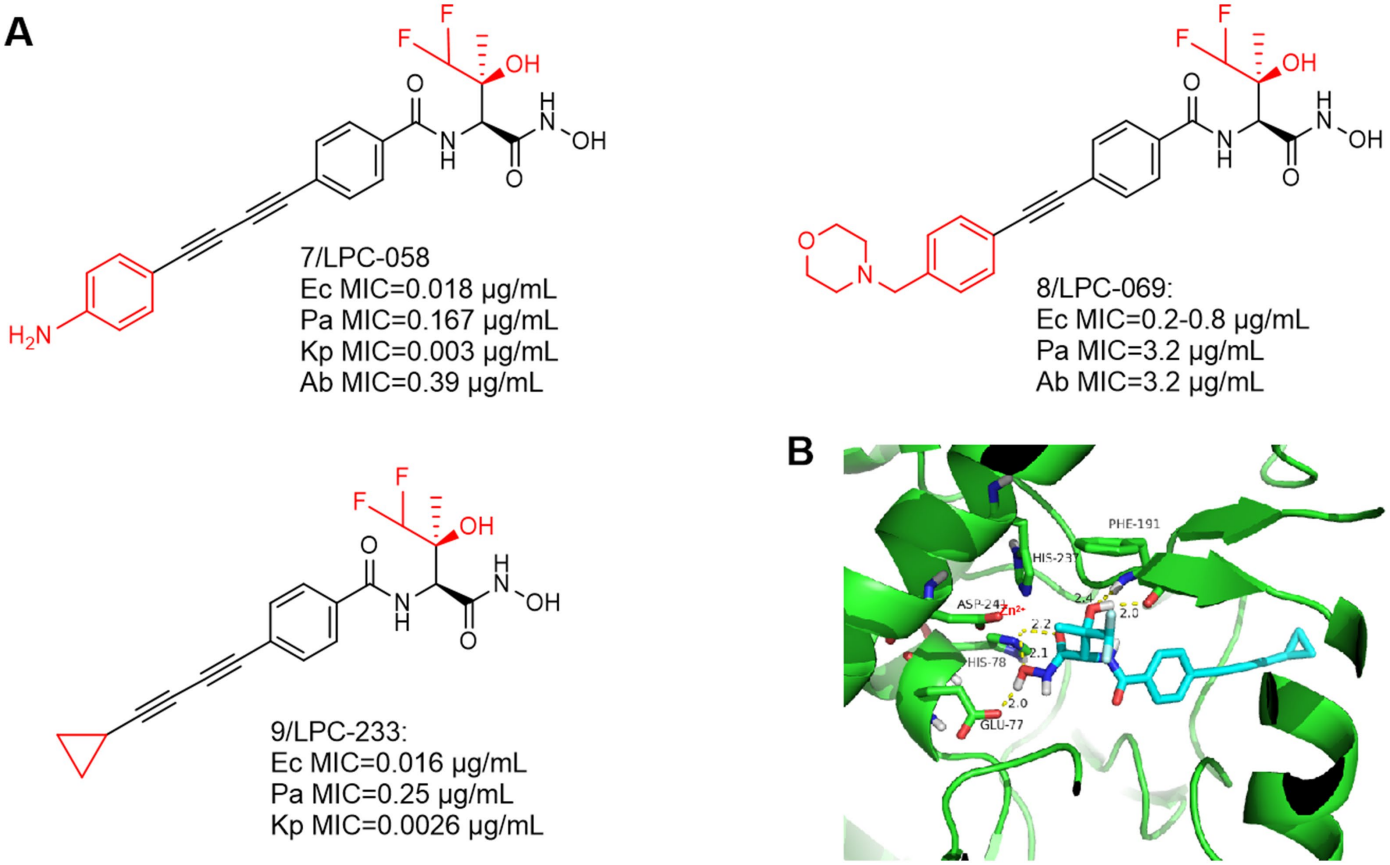
**(A)** Difluoromethyl-L-threonine hydroxamic acid-based hydroxamic acid inhibitor developed on the basis of CHIR-090; **(B)** The binding mode of LPC-233 in complex with *P. aeruginosa* LpxC. (PDB code: 7DEM).

The most notable feature of such compounds is the introduction of a difluoromethyl fragment in the **P2** region. As shown in Figures 4A,B, a compound with broad-spectrum antimicrobial activity, LPC-058 (Compound **7**), was reported in 2016, which was effective against *E. coli*, *P. aeruginosa*, and against *A. baumannii*. It is one of the few compounds with activity against *A. baumannii* (the difluoromethyl fragment may have increased the ability to cross the lipid barrier), but there were significant toxic side effects of this compound at high doses (Lemaître et al., 2017; Titecat et al., 2016). Notably, LPC-058 was very active against *Y. pestis*, with a MIC similar to that of ciprofloxacin (0.03 μg/mL). Another compound with broad-spectrum antimicrobial activity, LPC-069 (Compound **8**), was reported in 2017. It was effective against *E. coli*, *P. aeruginosa*, and against *A. baumannii*, and the researchers found no significant toxicities under high dose conditions. In contrast to LPC-058, LPC-069 was not toxic in mice and was capable of curing plague.

This compound is slightly different from LPC-058 at the hydrophobic end, Jinshi Zhao et al. replaced the terminal aniline structure of LPC-058 with a cyclopropargyl structure, which may effectively increase the water solubility of the compound and may reduce its toxicity. The study demonstrated that when LPC-233 (Compound **9**) was administered via the intravenously (IV), intraperitoneally (IP), or orally (PO) routes, it effectively eliminated bacterial infections in a variety of murine models (Zhao et al., 2023).

At the highest dose (40 mg/kg per 12 h), LPC-058 exhibited side effects such as diarrhea, leukocytosis, and hepatotoxicity (Lemaître et al., 2017). The toxicity of LPC-058 may be related to the aniline moiety. Compared to LPC-058, in a 7-day repeated-dose toxicity study in rats, LPC-233 was safely administered for seven consecutive days at a maximum dose of 125 mg/kg every 12 h (250 mg/kg per day). Most notably, it had high oral bioavailability (73% under fasting conditions) and an oral dose of 40 mg/kg per 12 h completely cleared bacterial infection in a murine UTI model (Zhao et al., 2023). These two factors may contribute to the unique safety profile of LPC-233. (1) LPC-233 contains a hydroxamic acid moiety but no amino group, thus attenuating the amino-associated toxicity observed in ACHN-975. (2) LPC-233 is highly bound to plasma, with binding fractions (fb) ranging from 96.4% in mice to 98.8% in humans. Limited amounts of unbound LPC-233 in plasma may also contribute to the lack of cardiovascular toxicity. The lack of plasma effect may be explained by the extremely tight binding affinity of LpxC for LPC-233 (Ki = 8.9 pM), as demonstrated by plasma protein binding experiments in the presence of LpxC, which competes with plasma proteins for binding of LPC-233, thereby effectively stripping bound LPC-233 from plasma proteins. Thus, the high percent plasma binding properties may provide a safety net to mitigate potential side effects of LPC-233.

It is worth noting that preclinical studies have shown a promising safety profile, with no detectable adverse cardiovascular toxicity in dogs at a dose of 100 mg/kg. These promising properties make LPC-233 an attractive candidate for further development in clinical trials. LPC-233 is being considered for Phase I clinical trials to investigate its safety and efficacy in humans.

#### 4.1.2 Alkyl sulfone-phenyl ring-hydroxamic acid-like LpxC inhibitors

**Figure 5:**
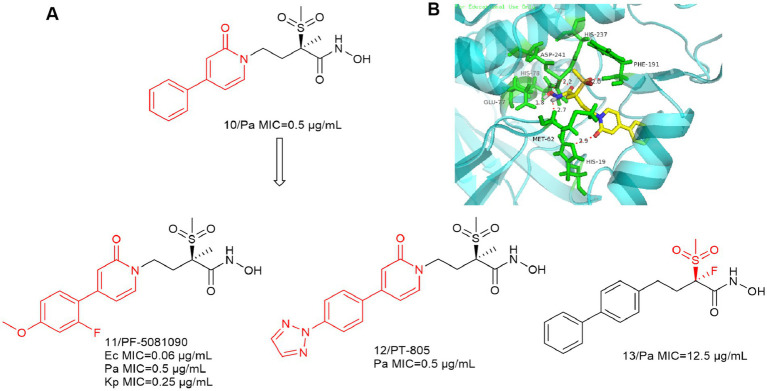
**(A)** Alkyl sulfone-phenyl ring-hydroxamic acid-like inhibitors; **(B)** The binding mode of Compound **10** in complex with *P. aeruginosa* LpxC (PDB code: 7DEM).

Since the first alkylsulfone hydroxamic acid inhibitor was reported and development, medicinal chemists have become increasingly interested in a variety of similar compounds. In Figure 5A, Compound **10** inhibitory activity against *P. aeruginosa* (PA01) was better than that of CHIR-090. The IC_50_ of compound **10** against the LpxC enzyme of *P. aeruginosa* was 3.6 nM, and further optimization produced compounds **11** and **12**. The addition of appropriately positioned substituents such as chlorine, fluorine and methoxy to the terminal substituent of the phenyl group of **10** was found to appropriately increase the potency of the compounds. As indicated, representative compound **11** exhibits broad-spectrum antimicrobial activity against Gram-negative bacteria, including *E. coli*, *P. aeruginosa* and *K. pneumoniae* (Tomaras et al., 2014).

As shown in Figure 5B, the intermolecular interactions associated with the Zn ion binding site are similar to those of CHIR-090. The alkyl sulfone structure in the **P2** region extends into the UDP pocket and forms a hydrogen bonding force with Phe191. In contrast to previous studies, the oxygen atom in the amide structure within the **P4** region forms a significant hydrogen bonding force with His19. This indicates that the structure may also function as a linker. It can be seen that the shorter hydrophobic structure as well as the absence of hydrophilic ends does not affect the antimicrobial activity of the inhibitors.

In 2020, Melanie Rodríguez-Alvarado et al. substituted F for Me to obtain compound **13** (Rodríguez-Alvarado et al., 2020). Theoretically, fluorine is closer to smaller hydrogen atoms than to larger methyl groups, and the substitution of fluorine for acidic H in the stereocenter to stabilize its conformation is expected to further enhance the activity of the compound. Unfortunately, it turned out in the end that the substitution of Me (or H) with F in the *α*-stereocenter of these LpxC inhibitors may not be very advantageous, and their activity is weaker than that of the lead compounds.

With the development of LpxC, such compounds are slowly fading into obscurity. The **P2** region of such compounds is an alkyl sulfone structure, which has a close interaction force with LpxC. The most important feature of this class of compounds is that their **P3** region is a non-alkyne structure, which provides a favorable reference for the subsequent development of LpxC inhibitors.

#### 4.1.3 Alkylsulfone-monoalkynyl-hydroxamic acid-like LpxC inhibitors

**Figure 6:**
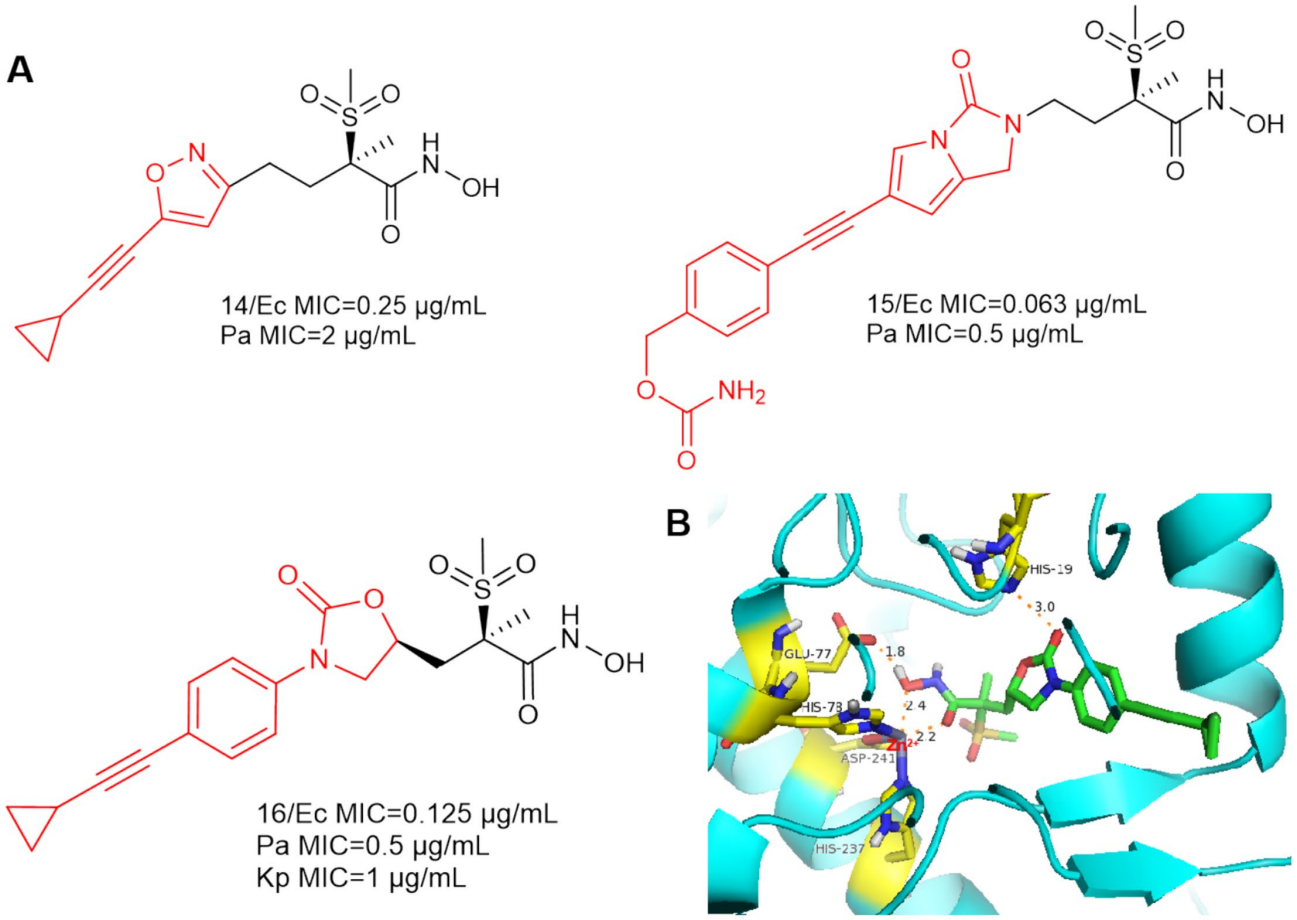
**(A)** Alkyl sulfone-monoalkynyl-hydroxamic acid-like inhibitors; **(B)** The binding mode of Compound **16** in complex with *P. aeruginosa* LpxC. (PDB code: 7DEM).

As shown in Figure 6A, an isoxazole analog compound **14** was reported in Novartis’ patent, while compound **15** was also included in the patent with superior anti-bacterial activities against *E. coli* (MIC = 0.063 μg/mL) and *P. aeruginosa* (MIC = 0.5 μg/mL) (Abdel-Magid, 2015).

In 2018, Patrick S. Lee et al. designed and synthesized compound **16** by computational scaffold jumping method (Lee et al., 2018). Due to the hydrogen bonding forces between His19 and the oxazolidinone structure, as well as the insertion of the alkyl sulfone into the UDP cavity. This may result in the loss of important hydrogen bonding interactions between the alkyl sulfone and the lysine residue (Lys 238), which is located near the catalytic site (Figure 6B). Compound **16** was potent against *P. aeruginosa* in a mouse model of neutropenic thigh infection and it showed strong antibacterial activity against Gram-negative bacteria such as *E. coli* and *K. pneumoniae*.

#### 4.1.4 Alkylsulfonyl diynyl hydroxamic acid LpxC inhibitors

**Figure 7:**
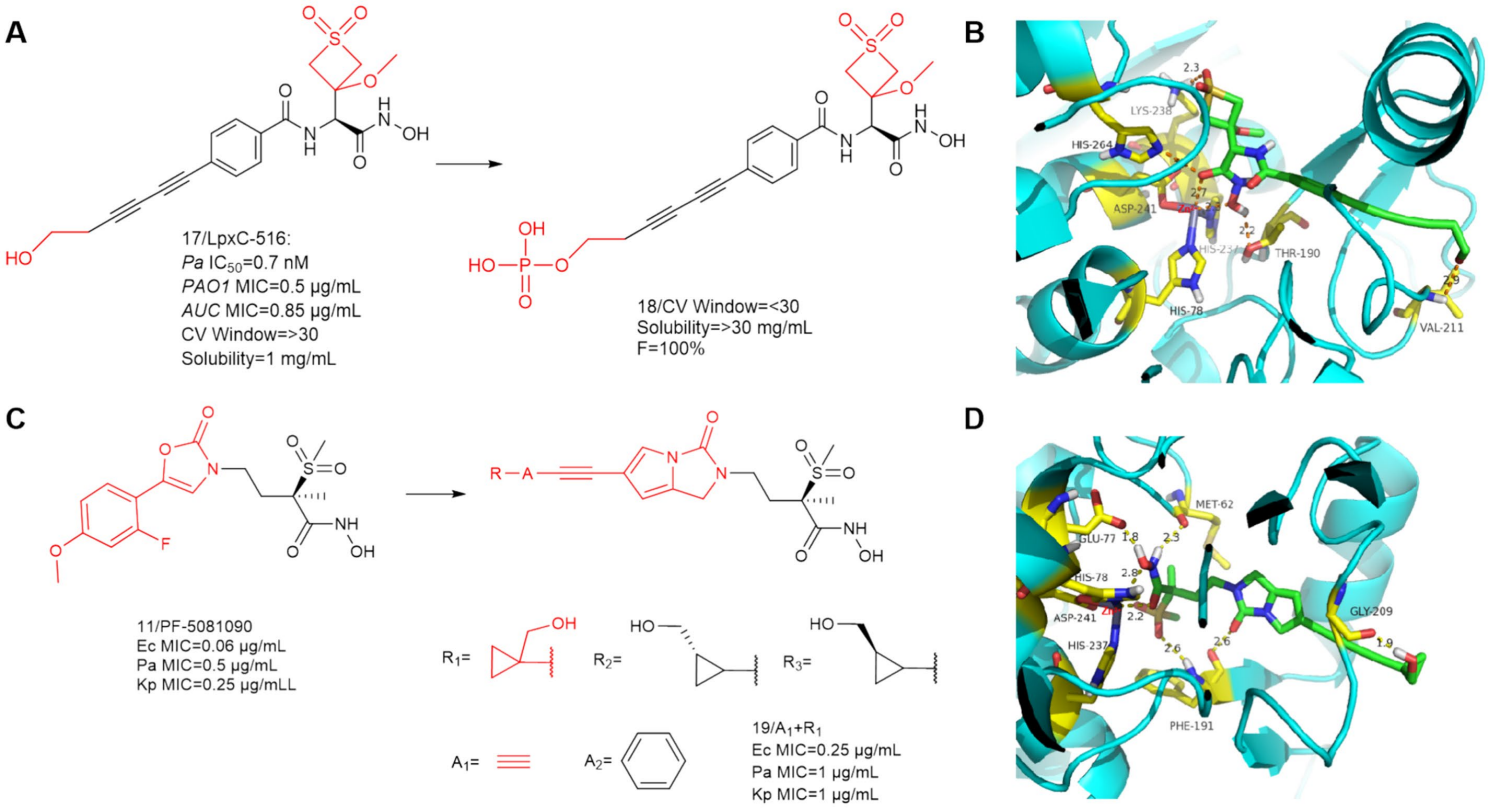
**(A)** Optimization of the hydrophobic terminus of LpxC-516; **(B)** The binding mode of compound **17** in complex with *P. aeruginosa* LpxC; **(C)** Optimization of PF-5081090; **(D)** The binding mode of compound **19** in complex with *P. aeruginosa* LpxC. (PDB code: 7DEM).

In 2019, Frederick Cohen et al. designed and developed the LpxC-516 (compound **17**) (Figure 7A), which has favorable activity against *P. aeruginosa* (Krause et al., 2019). As shown in Figure 7B, similar to previous findings, there is a significant hydrogen bonding interaction between the alkyl sulfone headgroup and the lysine residue (Lys 238), which is located near the catalytic site adjacent to the methyl ether extending into the UDP pocket. The difference is that there is also a Thr190 at the hydroxamic acid group position that forms an important hydrogen bonding force with the hydroxyl group. The hydroxyl group at the hydrophobic terminus does not fully extend into the solvent-exposed region, but rather there is a hydrogen bonding force with the Val211 of the LpxC protein, which binds it tightly to the protein. LpxC-516 hydroxamic acid salt is unstable at elevated pH and does not increase in solubility at lower pH, Frederick Cohen et al. made it into a phosphate precursor (Compound **18**), which was successful in increasing the drug solubility of the precursor drug. Its phosphate will be rapidly converted to the active drug, with a half-life of 2.4 min and 100% bioavailability of the precursor drug. Unfortunately when tested in a rat CV safety assay, the final lead prodrug compound showed unacceptable CV toxicity, which contrasts with the results obtained when the parent compound was given in the HPCD formulation, the reason for this discrepancy remains unclear (Krause et al., 2019).

Surivet et al. (2020), obtained compound **19** by structural optimization on the basis of PF-5081090 (Panchaud et al., 2019; Surivet et al., 2020). The UDP pocket alkyl sulfone structure and the hydroxamic acid structure were retained, and a hydrophobic channel for imidazolone was introduced in the **P3** region. In previous SAR studies on CHIR-090 analogs, inhibitors with small alkyne units in the threonyl hydroxamic acid series achieved broad and potent antibiotic activity. As shown in Figure 7C, Compound **19** showed good activity against a wide range of Gram-negative bacteria.

SAR study of compound **19** indicated that when A group is a benzene ring, all these derivatives were potent LpxC inhibitors, as evidenced by their strong binding affinity for the LpxC enzymes of Ec (ΔTm = 11–12 K) and Pa (ΔTm = 13–14 K). The R group has a great influence on the antimicrobial activity of the bis-alkynyl (A group is alkynyl) compounds compared to the phenylethynyl (A group is a benzene ring) compounds. The compounds of this series (A group is alkynyl) exhibited good solubility (>350 μg/mL) at pH = 7, whereas their respective phenyl analogs (A group is a benzene ring) exhibited limited solubility (ranging between 7 and 112 μg/mL) at the same pH (Surivet et al., 2020).

As shown in Figure 7D, the overall conformation of the compound is altered due to the introduction of the five-membered heterocycle, the binding mode for the binding site of its hydroxamic acid to zinc ions is slightly different from that of the methylsulfone-based hydroxamic acid LpxC inhibitors. The binding mode here is identical to that of CHIR-090, and in addition to the three amino acids that immobilize the zinc ion, there are also two amino acids, Met62 and Glu77, as in CHIR-090, that form important hydrogen bonding interactions with the hydroxamic acid. In addition to this, unlike other methylsulfone hydroxamic acid LpxC inhibitors, there is also a hydrogen bonding force that Phe191 shares with methylsulfone and pyrrolobenzimidazolone. The cyclopropyl ring occupies a shallow hydrophobic side pocket in both fine conformations, with the hydroxymethyl group pointing toward the solvent in the opposite direction of the cyclopropyl unit (in both conformations). The hydrophobic terminus extends into the solvent-exposed region, and due to the bending of the terminal hydroxyl group, compound **19** has hydrogen bonding forces with Gly209 of this protein. Similar to all related derivatives, the methylsulfone hydroxamic acid ester head and the pyrroloimidazolone core bind to *Pa* LpxC, and the bis-alkynyl metal needle fills the substrate channel, projecting the moiety R onto the edge of the protein.

#### 4.1.5 Methylsulfonamide non-alkynyl hydroxamic acid LpxC inhibitors

**Figure 8:**
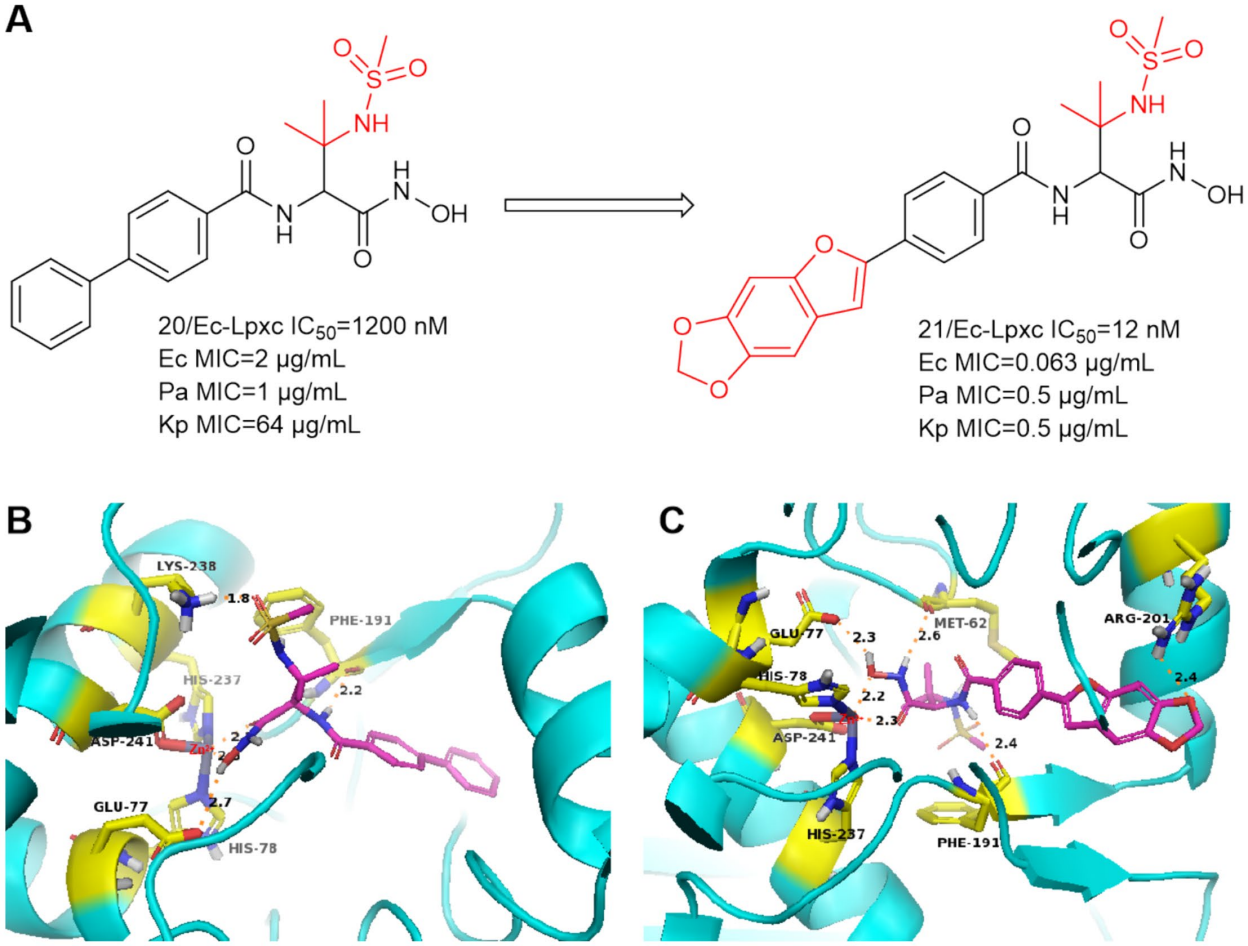
**(A)** Methanesulfonamide non-alkynyl hydroxamic acid LpxC inhibitors; **(B)** The binding mode of compound **20** in complex with *P. aeruginosa* LpxC; **(C)** The binding mode of compound **21** in complex with *P. aeruginosa* LpxC. (PDB code: 7DEM).

In 2016, Tetsuya Kawai et al. focused on the improvement of enzyme inhibition and antimicrobial activity to optimize sulfonamide-based non-alkyne LpxC inhibitors (Kawai et al., 2017). Compound **20** showed moderate enzyme inhibitory activity against *P. aeruginosa* and *in vitro* antimicrobial activity against a wide range of Gram-negative bacteria (Figure 8A). As shown in Figure 8B, the nitrogen of sulfonamide appears to have the potential to utilize the critical Lys 238 hydrogen bond in *P. aeruginosa* LpxC. In Figure 8A, compound **21** with 2-arylbenzofuran as a hydrophobic moiety was found to have very excellent *in vitro* antimicrobial activity against a wide range of Gram-negative bacteria (*Ec*, *Pa* and *Kp*) and it has a good slow release activity. The reason for this may be that the introduction of 2-arylbenzofuran in the **P4** region led to a change in the overall conformation of compound **21** (Figure 8C). The binding pattern of its hydroxamic acid to zinc ions is similar to that of CHIR-090, and the tailed furan structure forms a hydrogen bonding interaction with Arg201. Compound **21** showed impressive antimicrobial activity and was a precursor for further exploration as an antimicrobial agent for non-alkyne LpxC inhibitors.

#### 4.1.6 Benzyloxyacetyl hydroxamic acid LpxC inhibitors

In 2019, Magdalena Galster et al. performed a divergent synthesis of the hydroxamic acid portion of benzyloxyacetyl hydroxamic acid to obtain phenylglycol derivatives exhibiting a variety of Zn^2+^ binding groups (Galster et al., 2019). For example, hydrazides, amides, sulfonamides, *o*-diols, thiols, thioesters, and hydroxypyridone derivatives were synthesized and tested for antimicrobial activity, and ultimately no compounds were found to be more prominently active.

**Figure 9:**
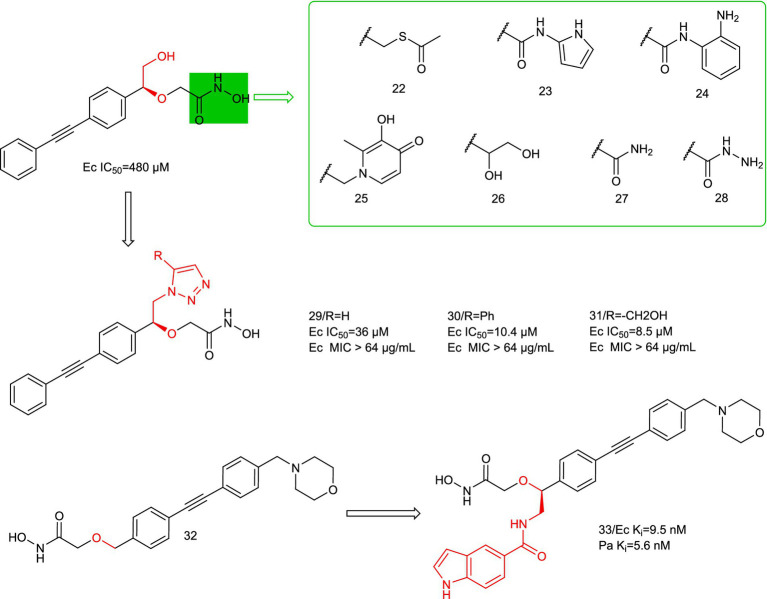
Benzyloxyacetyl hydroxamic acid LpxC inhibitors.

In 2020, Katharina Hof et al. performed a divergent synthesis of the hydroxyl portion of benzyloxyacetyl hydroxamic acid to obtain a series of triazole-based LpxC inhibitors, and ultimately found that the triazole portion was a suitable functional group (Figure 9, compounds 29–31) (Hoff et al., 2020). This could address additional binding sites for LpxC, but in *in vitro* antimicrobial assays, the ability of its series of compounds to resist *E. coli* was not quite as prominent.

In 2024, Sebastian Mielniczuk et al. developed a benzyloxyacetohydroxamic acid LpxC-based inhibitor, it specifically targets the UDP binding site of the enzyme (Mielniczuk et al., 2024). Compound **33** showed high affinity for the LpxC enzymes of *E. coli* and *P. aeruginosa*, and it exhibited low MIC values for *E. coli* (*E.coli* BL21 (DE3) (1–4 μg/mL) and *E. coli* D22 (0.031 μg/mL)). Compound **33** has potential off-target effects on several mammalian zinc-dependent enzymes (MMP 1–3 and TACE), suggesting good selectivity for LpxC. Compound **33** has low plasma stability in mouse plasma and thus needs to be improved in further optimization steps. Structural optimization of the **P2** region alone may achieve an improvement over LpxC inhibitors.

#### 4.1.7 C-furanoside-based hydroxamic acid LpxC inhibitors

**Figure 10:**
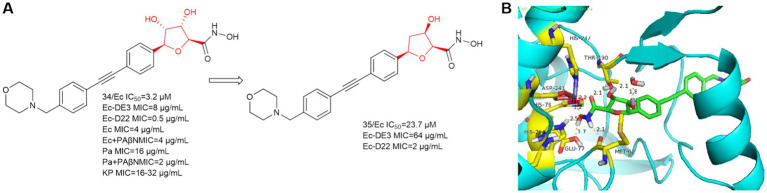
**(A)** C-furanoside analogs of hydroxamic acid LpxC inhibitors; **(B)** The binding mode of compound **34** in complex with *P. aeruginosa* LpxC. (PDB code: 7DEM).

In 2021, Alexander Dreger et al. obtained a series of C-furanoside LpxC inhibitors in a chiral pool synthesis using D- and L-xylose as starting materials (Dreger et al., 2021). This is similar to a class of glycoside-containing LpxC inhibitors synthesized by Oddo et al. in 2012, with R and S conformational differences only at the hydroxyl carbon atom of the five-membered sugar ring. Compound **34** exhibited 2,3-trans- and 4,5-trans-configurations and was the most active stereoisomer of the furanoside series studied (Figure 10A). As in Figure 10B, the hydroxyl group on the furan of compound **34** extends into the cavity and forms a hydrogen bond with water. At the site where the hydroxamic acid binds to the zinc ion, in addition to the three amino acids that immobilize the zinc ion, four amino acids, Glu77, His264, Met62, and Thr190, form strong hydrogen bonds with the hydrogen atom of the hydroxyl group of the hydroxamic acid, the hydrogen atom of the hydroxyl group, the hydrogen atom of the amino group, and the oxygen atom of the carbonyl group, respectively. This allows the protein to strongly immobilize the compound **34** in the protein. In the same year, Alexander Dreger et al. designed a series of monohydroxy-substituted tetrahydrofuran derivatives LpxC inhibitors based on compound **34** (Dreger et al., 2021). The most promising LpxC inhibitor among them is 3-hydroxytetrahydrofuran derivative **35**, but its inhibitory activity is inferior to compound **34**.

Notably, in experiments with and without the efflux pump inhibitor PAßN, compound **34** was tested against *P. aeruginosa* ATCC-27853 and a significant 8-fold reduction of the MIC was observed in the presence of PAßN (three serial dilution steps). In a further optimization step, the bacterial permeability of the C-glycoside LpxC inhibitor needs to be increased.

#### 4.1.8 Tetrahydropyran-type hydroxamic acid LpxC inhibitors

**Figure 11:**
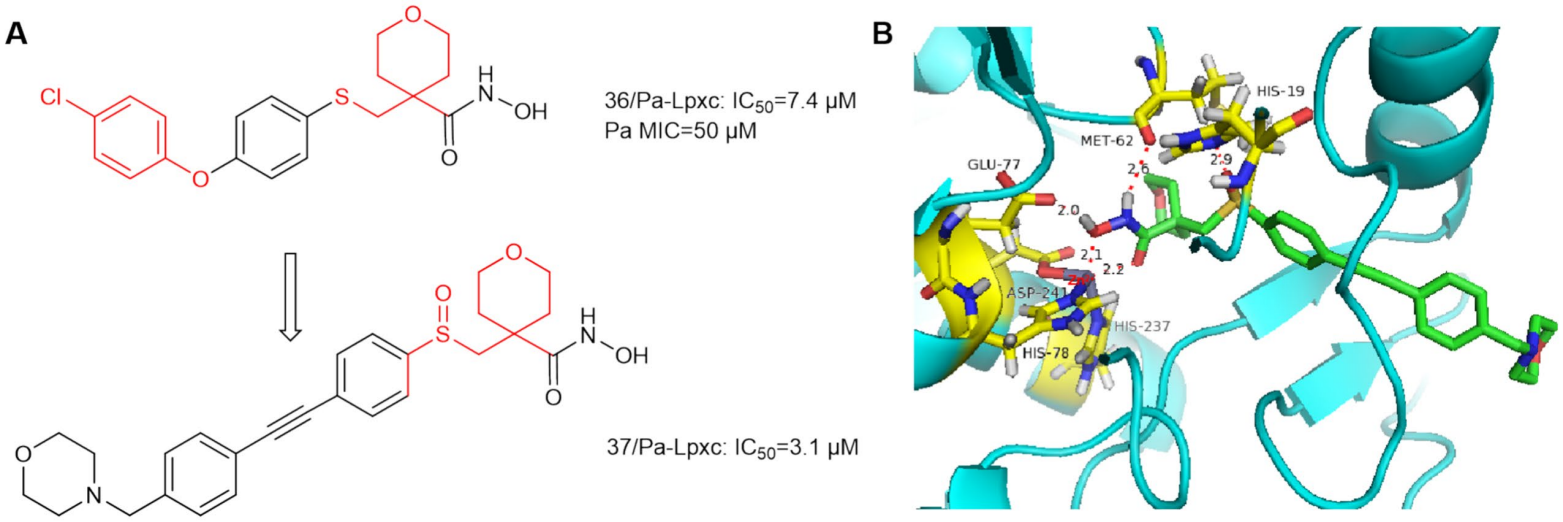
**(A)** Tetrahydropyran-like hydroxamic acid LpxC inhibitors; **(B)** The binding mode of compound **36** in complex with *P. aeruginosa* LpxC. (PDB code: 7DEM).

In 2014, AstraZeneca Murphy Benenato et al. screened the library of hydroxamic acid compounds and obtained compound **36** (Figure 11B), which is a derivative of the matrix metalloproteinase (MMP-2, −9, −8, −13) inhibitor JR-130830 (Murphy-Benenato et al., 2014). Compound **36** has activity against *P. aeruginosa*, but its activity against *E. coli* ARC523 is weak (MIC>200 μmol/L). Using compound **36** as the lead compound and retaining the tetrahydropyran ring and optimize the hydrophobic region. As shown in Figure 11B, the sulfoxide structure of compound **37** formed a hydrogen bond with His19 leading to a higher affinity of compound **37** for the protein. The hydrophobic end of compound **37** extends further into the hydrophobic channel, and shows increased activity against *E. coli* compared to compound **36**. Finally although it exhibited low molar potency against *P. aeruginosa* LpxC, its effective MMP inhibition was retained, which increased off-target effects. Since then, such compounds have not been mentioned.

#### 4.1.9 Unnatural amino acid-based hydroxamic acid LpxC inhibitors

**Figure 12:**
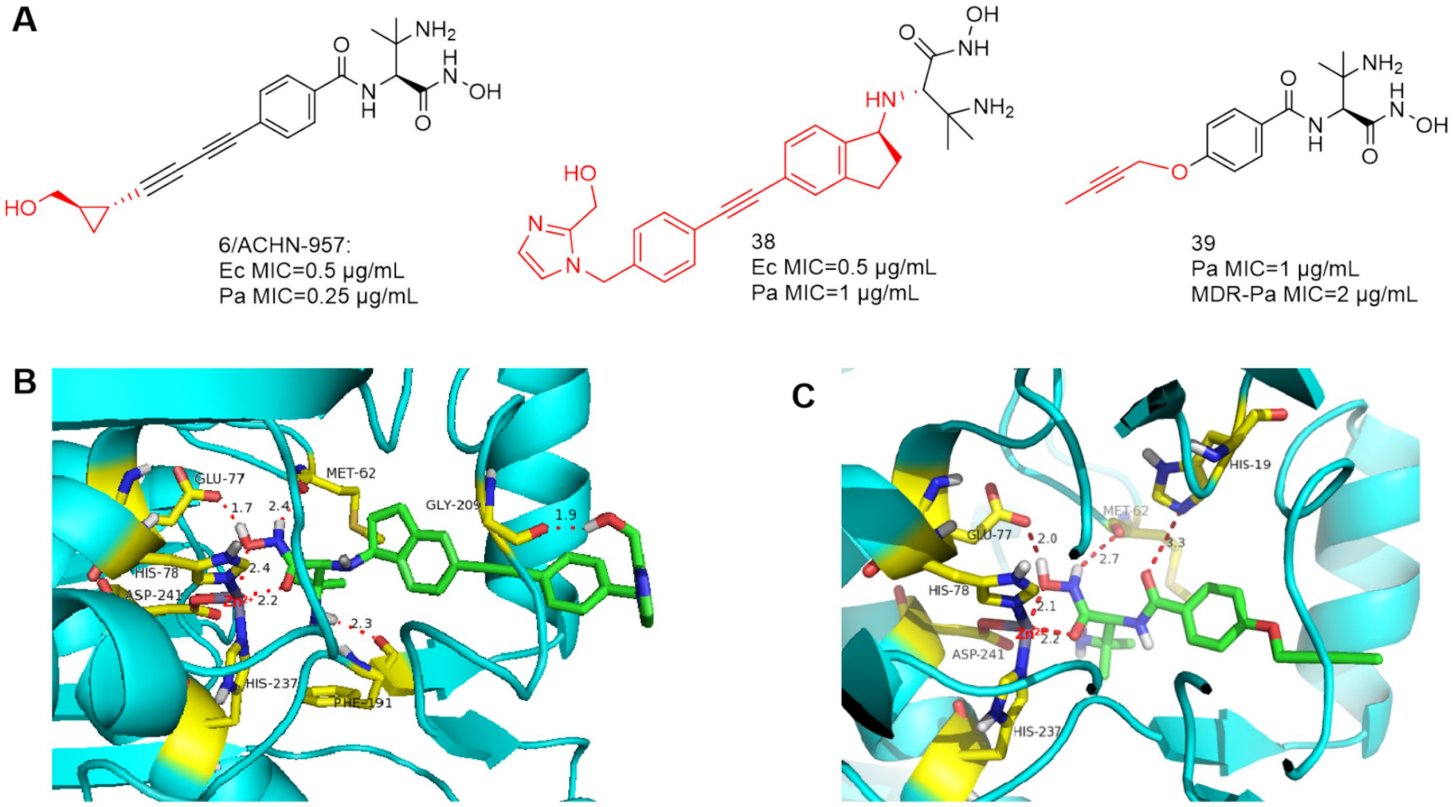
**(A)** Unnatural amino acid-based hydroxamic acid LpxC inhibitors; **(B)** The binding mode of compound **38** in complex with *P. aeruginosa* LpxC; (PDB code: 7DEM). **(C)** The binding mode of compound **39** in complex with *P. aeruginosa* LpxC. (PDB code: 7DEM).

Based on ACHN-975, compounds **38** and **39** (Figure 12A) were optimized and synthesized, respectively. In 2017, Jing et al. reported that the linker is a dicyclic analog, representing compound **38** with good broad-spectrum antimicrobial activity (Zhang et al., 2017). As in Figure 12B, Phe191 is 2.3 Å away from the amino group of compound **38** and forms a hydrogen bond, and the terminal hydroxyl group bends inward and forms a hydrogen bond with Gly209. Acute tolerability problems observed in phase IV rats prevented its entry into clinical trials due to its intravenous acute toxicity and inhibition of sodium channel site II (97%, 25 μmol/L, IC_50_ = 400 nmol/L).

Based on ACHN-975, in order to improve the water solubility and reduce the toxicity, Piizzi et al. of Novartis optimized the bis-alkynyl structure of the **P4** region of ACHN-975. Compounds **39** was effective against MDR *P. aeruginosa*, however, its antimicrobial activity is no more desirable than ACHN-975 (Piizzi et al., 2017). As shown in Figure 12C, in the important Zn ion binding site and UDP pocket region, compound **39** interacts with the LpxC protein of *P. aeruginosa* similarly to CHIR-090, and the alkynyl propoxy occupies the hydrophobic cavity and extends into the solvent-exposed region.

### 4.2 Non-hydroxamic acid LpxC inhibitors

#### 4.2.1 Phosphoric LpxC inhibitors

**Figure 13:**
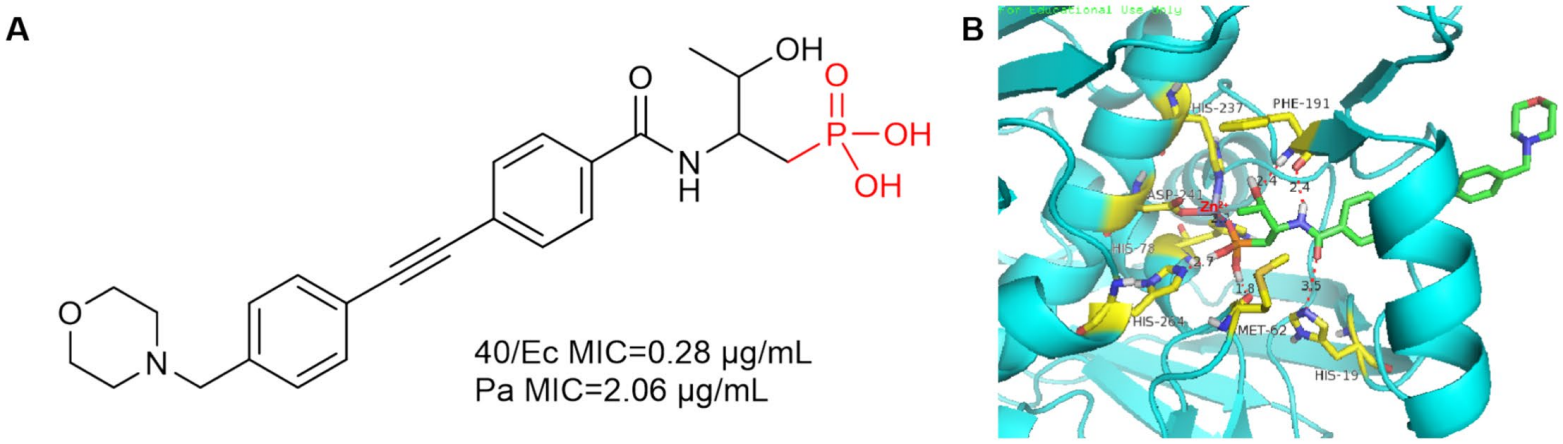
**(A)** Phosphate Lpxc inhibitor; **(B)** The binding mode of compound **40** in complex with *P. aeruginosa* LpxC. (PDB code: 7DEM).

In 2016, Yucheng Wang et al. introduced the phosphate group into the **P1** region of CHIR-090 to obtain compound **40**, which showed comparable inhibition of *E. coli* and *P. aeruginosa* to CHIR-090 (Figure 13A). As in Figure 13B, Met62 and His264 are 1.8 Å and 2.7 Å away from the hydroxyl group of phosphoric acid, respectively, and form hydrogen bonds with it. Phe191 forms a hydrogen bond with the hydroxyl group and amide of threonine, and due to the introduction of the phosphate group, His19 is more distant from the amide of compound **40** (3.5 Å) relative to CHIR-090 (3.3 Å).

#### 4.2.2 N-hydroxyformamide LpxC inhibitors

**Figure 14:**
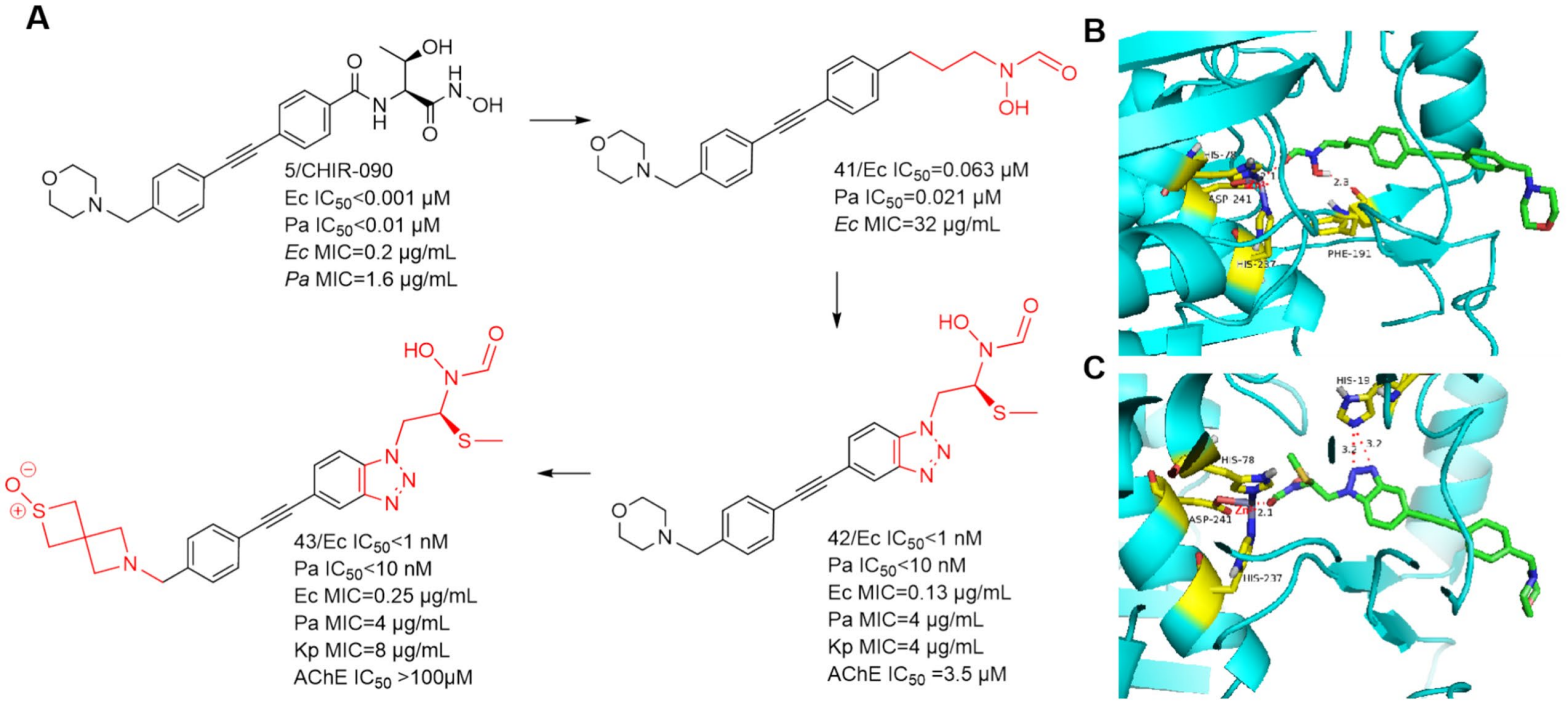
**(A)** *N*-hydroxyformamide-based LpxC inhibitors; **(B)** The binding mode of compound **41** in complex with *P. aeruginosa* LpxC; (PDB code: 7DEM). **(C)** The binding mode of compound **42** in complex with *P. aeruginosa* LpxC. (PDB code: 7DEM).

In 2020, Takeru Furuya et al. used CHIR-090 as a lead compound, focusing on the hypothesis that the *N*-hydroxycarboxamide warhead could overcome the previous toxicity problems of LpxC inhibitors by substituting the terminal hydroxamic acid (Furuya et al., 2020). As shown in Figure 14A, Compound **41**, which was just successfully obtained, was inhibitory to the LpxC enzymes of *E. coli* and *P. aeruginosa*, and showed comparable viability to *E. coli* ATCC-25922. As in Figure 14B, the binding site of compound **41** to the zinc ion is the carbonyl structure of the *N*-hydroxyformamide, and then Phe191 forms a hydrogen bond with the hydroxyl group of compound **41**. The use of benzotriazole structure to replace the benzene ring structure effectively enhanced the activity, the reason for this is that the benzotriazole forms a strong hydrogen bond with the imidazole group of His19 (Figure 14C). A potent and effective LpxC inhibitor against Gram-negative bacterial infections was obtained (compound **42**), but it had a strong inhibition rate of AChE and nearly 2/3 of the mice died in the safety evaluation of the hemodynamic test in rats. To solve this problem, the morpholine tail was replaced with known morpholine bioelectronic equivalents and structurally related heterocycles. Compound **43** stood out in terms of lower rat plasma protein binding (9% unbound compared to 3% for compound **42**) and weaker AChE inhibition (>100 μM compared to 3.5 μM for compound **42**). In safety evaluations of hemodynamic tests, rats receiving compound **43** showed hypotension with plasma concentrations of 11–16 μg/mL and did not rebound during the recovery period. Despite acceptable PK and *in vitro* safety data, the compound **43** showed unacceptable swelling effects in rat studies.

#### 4.2.3 2-(1-S-hydroxyethyl) imidazoles LpxC inhibitors

**Figure 15:**
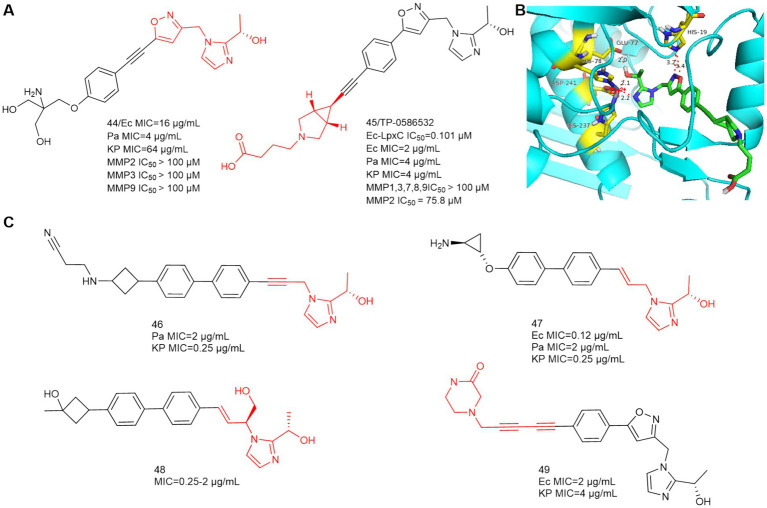
**(A)** 2-(1-S-hydroxyethyl)imidazole-based LpxC inhibitors; **(B)** The binding mode of TP 0586532 in complex with *P. aeruginosa* LpxC; (PDB code: 7DEM); **(C)** Other 2-(1-S-hydroxyethyl)imidazole-based compounds disclosed in patents.

In 2020, Yousuke Yamada et al. synthesized 2-(1-*S*-hydroxyethyl) imidazole derivatives exhibiting low nanomolar inhibition of LpxC, which are virtually unaffected by the presence of albumin (Yamada et al., 2020). In 2021, Fumihito Ushiyama et al. designed TP 0586532 to be effective against carbapenem-resistant *K. pneumoniae* without posing cardiovascular risks (Ushiyama et al., 2021). TP 0586532 shows potent activity against a broad range of carbapenemase gene-positive strains, including blaNDM, blaKPC, blaVIM, blaOXA and blaIMP (Fujita et al., 2022). TP 0586532 has excellent activity against mucin-resistant strains, ESBLs-producing *E. coli*, and carbapenem-resistant *K. pneumoniae*. As shown in Figure 15A, the IC_50_ of TP 0586532 for human MMPs is more than 700 times higher than that for E.coli LpxC, so TP 0586532 has excellent selectivity for human MMPs.

In Figure 15B, the N and O of 2-(1-S-hydroxyethyl) imidazole chelate Zn ions by hydrogen bonding forces, which is the same as compound **44**. The five-membered oxazole heterocycle of TP 0586532 then forms a hydrogen bond with His19, the hydrophobic structure is inserted into the hydrophobic cavity in a compliant manner, and the carboxylic acid structure at the hydrophobic terminus bends toward the side of the LpxC protein, but there is no associated hydrogen-bonding force with the amino acid residue in question.

*In vitro* LPS level assays demonstrated that TP 0586532 exerted antimicrobial activity by inhibiting LPS synthesis and that TP 0586532 reduced total LPS levels in a concentration-dependent manner (Fujita et al., 2022). TP 0586532 enhances the antimicrobial activity of various antibiotics (especially meropenem) against *Enterobacteriaceae*, including CRE (Yoshida et al., 2022). The mechanism of action is related to increasing bacterial cell membrane permeability and facilitating the entry of meropenem into the cell. This study demonstrates that meropenem in combination with TP 0586532 has therapeutic potential for the treatment of severe CRE infections, including strains with high levels of resistance to meropenem. This also suggests that combination therapy with antimicrobials of different antimicrobial mechanisms can compensate for the deficiency of LpxC inhibitors. TP 0586532 exerts potent efficacy in mouse models of systemic infection and pneumonia caused by gram-negative pathogens (Fujita et al., 2022; Ushiyama et al., 2021). TP 0586532 is expected to be the most promising LpxC inhibitor to enter clinical trials after ACHN-975.

Small molecule LpxC inhibitors with 2-(1-*S*-hydroxyethyl) imidazole as the active center have become a hotspot for optimization of LpxC inhibitors. As shown in Figure 15C, in 2022, Forge Therapeutics’ patent disclosed that compounds **46** and **47** could be used to treat bacterial infections, particularly pneumonia, both compounds had better antimicrobial activity than TP 0586532 (WO2022173756; WO2022173758, Min et al., 2022a,b). In 2024, Blacksmith Pharmaceuticals’ patent disclosed that compound **48** could be used to treat *Pseudomonas aeruginosa* infections, lung disease, and infectious pneumonia (WO2024036170, David et al., 2024). In 2024, researchers from Shanghai Angry Pharmaceuticals and Zhejiang Haizheng Pharmaceuticals introduced an aromatic acetylene group in the **P4** region to obtain compound **49** for treating Gram negative bacterial infections, of which oral bioavailability is 89.4% (WO2024083177, Xiangui et al., 2024).

## 5 Conclusion

With the emergence and rapid increasing of multidrug-resistant Gram-negative bacterial strains, the development of novel antibiotics directed against the previously unexploited targets is urgently needed (Alanis, 2005). Lipid A forms the outer monolayer of the outer membrane of most Gram-negative bacteria (Kim et al., 2016; Raetz et al., 2007), the LpxC-catalyzed reaction is an irreversible and essential step in lipid A biosynthesis (Jackman et al., 2000; Löppenberg et al., 2013). Thus, LpxC has become an attractive target for the structure-based drug design. Currently, there are LpxC inhibitors showing promising efficacy in infected animal models (Kalinin and Holl, 2017; Zhao et al., 2023). However, except the discontinued ACHN-975, no other LpxC inhibitors have entered clinical trials.

The existing LpxC inhibitors can be divided into two main categories according to the structure, namely hydroxamate inhibitors and non-hydroxamate inhibitors. Due to the potential toxicity of hydroxamic acid group in the metabolism of organisms, great efforts have been made in recent years to develop non-hydroxamic acid inhibitors, exemplified by 2-(1-S-hydroxyethyl) imidazole compound TP 0586532. The design and further optimization of LpxC inhibitors should focus on the screening using computer-aided drug design, e.g., through computational methods utilizing multiple crystal structures to aid in the identification and synthesis of novel LpxC inhibitors, such as compound **16** (Lee et al., 2018). Furthermore, multi-target drug design strategy should also be taken into account when designing novel LpxC inhibitors against a wide range of Gram-negative bacteria. For example, macrolones, the synthetic macrolide derivatives with a quinolone side chain, efficiently inhibit both the ribosome and DNA topoisomerase *in vitro* (Aleksandrova et al., 2024). In this paper, we summarize the research and development of LpxC inhibitors from 2014 to 2024, expecting to prompt the approval and application of LpxC inhibitors in clinical practice.
